# audit_cc: A Stata command for the analysis of matched case-control audits of cervical cancer screening

**DOI:** 10.1016/j.imu.2023.101182

**Published:** 2023

**Authors:** Milena Falcaro, Peter Sasieni, Alejandra Castañon

**Affiliations:** Cancer Prevention Group, School of Cancer & Pharmaceutical Sciences, King's College London, UK

**Keywords:** audit_cc, Audits, Cervical screening, Matched case-control study, Monitoring, Stata

## Abstract

**Background and objectives:**

Cervical screening programmes are crucial for the early diagnosis and prevention of cancer of the cervix. Regular auditing is vital for ensuring that these programmes achieve their full potential and meet their objectives in practice. Unfortunately, the time and skills required for the statistical analysis of the data collected are often important limiting factors. Comparisons across countries and over time have also been particularly difficult due to a lack of standardized definitions and methodology. We aimed to overcome these problems.

**Methods:**

Using the statistical software Stata, we developed a new command called audit_cc for the analysis of matched case-control audits of cervical cancer screening. Analyses are reported for two measures of screening history: time since last test and time since last negative test.

**Results:**

The command carries out the data manipulation which is required for the analysis and allows to save the resulting data set in an external file for further investigations. It promotes consistent evaluations of screening programmes over time and across studies and facilitates the creation of automatic publication-quality reports, which are especially useful in the context of routine audits.

**Conclusions:**

audit_cc is a valid tool that not only simplifies the analysis and reporting of cervical screening audits but also allows meaningful international comparisons. Although it is specific for cervical cancer, it can be seen as an example of how the standardisation of exposure definitions and key methodological issues can enable consistent and comparable evaluations of screening programmes across different countries and settings.

## 1 Introduction

Cervical screening programmes are vital for the early diagnosis and prevention of cancer of the cervix. Their aim is to detect precancerous lesions that can be treated before they develop into invasive cancer, contributing in this way to a reduction of incidence and mortality from this type of cancer. Regular audits of screening programmes are however essential because they help not only to assess if the objectives are reached but also to identify areas of good practice and where improvements might be needed [1,2]. Matched case-control studies are, if well designed and conducted, a powerful and efficient tool in this context [3,4] as they do not require information on all individuals screened or invited to screening but only data for those diagnosed with cancer (cases) and a sample of individuals (controls) who did not develop cancer and are matched to the cases on a set of characteristics.

Castañon and colleagues [5] carried out a systematic review of case-control studies evaluating the effect of screening on cervical cancer incidence and concluded that standard measures of exposure to screening are needed to facilitate international comparisons and for a consistent evaluation of cervical cancer screening programmes over time and across studies.

In this paper we describe a new Stata [6] command, called audit_cc, for the analysis of matched case-control audits of cervical screening programmes. Its aim is to promote the use of standard definitions of screening history exposure, allowing for consistent evaluation of cervical screening programmes and their international comparisons. It also greatly simplifies the creation of publication-quality automatic reports, which can be especially convenient for routine audits.

## 2 Methods

In matched case-control studies individuals with a certain condition of interest (i.e. cases) are matched with one or more individuals without the condition (i.e. controls) on the basis of a set of variables (e.g. age, gender, geographical area). The “outcome” is a binary variable equal to 1 for cases and 0 for controls and the matching is commonly accounted for by using conditional logistic regression, also known as a fixed-effects logit model for panel data. This model is similar to an ordinary (unconditional) logistic regression except that data are grouped and conditional likelihood is used instead [7,8]. Ordinary logistic regression can be used to analyse stratified data by incorporating a separate intercept parameter for each stratum (and omitting the overall intercept). However, this approach yields inconsistent estimates when the numbers in each stratum are small. When there is just one control matched to each case, adding a dummy variable for each matched pair is particularly problematic. Conditional logistic models overcome these difficulties by conditioning on the marginal results (i.e. the number of cases and of controls and the numbers with different levels of exposure) within each matched set so that the estimation of the group-specific intercepts is no longer necessary [[7], [8], [9], [10]].

In case-control studies of cancer screening the cases are women who had a diagnosis of cancer and the controls are usually matched or approximately matched by date of birth (and hence age) and possibly by geographical area, although matching on other variables is also possible where data availability allows it. Cases and controls are then compared in terms of their screening history exposure. The most straightforward way to specify exposure when there is a paucity of more specific screening history, and/or test results are not available, is to consider the time elapsed since the last test. This is hereafter referred to as the time-since-last-test exposure and is defined as the time between the date of diagnosis for cases or the corresponding index date (i.e. the matched case's date of diagnosis) for controls and the first eligible test prior to that date. Analyses where the exposure is defined as ever having had a test, regardless of the result, are used to estimate the effect of participating in screening. If the attention is restricted to the most recent test with a negative result (i.e. a time-since-last-negative-test exposure) then the focus is on the evaluation of how long individuals remain at lower risk of cancer following a negative screen [5].

Studies evaluating cervical screening programmes should distinguish between screening and diagnostic tests because only the former can be used to prevent cancer from occurring or to diagnose it before it becomes symptomatic. The period of time during which premalignant alterations have occurred but have not yet developed into invasive cancer is known as the pre-invasive detectable phase (PIDP), whereas the subsequent interval where the malignancy is present but does not produce symptoms is called the occult invasive phase (OIP) of tumour progression [11]. By definition, it is only during the PIDP period that screening can lead to the detection and treatment of lesions to prevent cancer. Unfortunately, in many jurisdictions, we do not know if tests were done for screening purposes or in response to symptoms. Sometimes the date and result of the test may help us to infer whether or not it was a diagnostic test. For example, tests that took place in proximity (generally up to 6 months) of a cancer diagnosis may be more likely to be diagnostic in nature and analysts may want to exclude them when deriving the time-since-last-test exposure [12]. When considering the time-since-last-negative-test exposure, Castañon and colleagues argued in favour of not excluding the tests taken close to diagnosis because a negative test during that phase is a false negative and capturing that is important for determining the longitudinal sensitivity of the screening test [5].

## 3 The audit_cc command

audit_cc fits a set of conditional logistic models for the analysis of matched case-control audits of cervical screening programmes. The command assumes that cases and controls have been (approximately or exactly) matched on age as part of the study design prior to analysis. This is because cervical screening is usually offered at different intervals depending on age and there is some evidence suggesting that the impact of screening differs by age. Matching on other variables is optional. Analyses are stratified by case's age group and reported for two screening exposure measures: (1) time since last test and (2) time since last negative test. In addition, users can easily generate automatic reports with results displayed in publication-quality tables and can save the manipulated data set used for the estimation of the conditional logistic models so that further analysis and the use of post-estimation commands (e.g. margins) are possible. audit_cc will be available from the Statistical Software Components (SSC) archive and it can be installed in Stata by typing “ssc install audit_cc, replace”.

### 3.1 Data structure

The audit_cc command requires the data to be organised in long format, with each screening test on a separate line. Women with no screening event must be included in the data set with a single record where the date and result of the test are missing. Each matched group must contain one case and one or more controls matched on age and, if necessary, other characteristics.

### 3.2 Syntax

audit_cc *depvar* [*if*] [*weight*] [, *options*]

where *depvar* is a variable that identifies the cases and controls: 1 corresponds to cases, whereas all other non-missing values are treated as controls. Note that this is different from how the clogit command would treat the dependent variable, i.e. non-zero and non-missing would indicate cases and zeros would represent controls. *fweights, iweights* and *pweights* are allowed but, since they apply to groups as a whole and not to individual observations, their values must be constant within each matched group.

#### 3.2.1 Options

**groupid(***varname***)** specifies the variable (either string or numeric) that uniquely identifies the matched groups. This option is required.

**id(***varname***)** specifies the variable (either string or numeric) that uniquely identifies the women. This option is required.

**caseddiag(***varname***)** identifies the variable containing the date of diagnosis (for cases) or the matched case's date of diagnosis (for controls). This option is required.

**casedob(***varname***)** names the variable for the case's date of birth (for the controls this is the date of birth of the matched case). Either this option or caseage(), but not both, must be specified.

**caseage(***varname***)** indicates the variable containing the age at diagnosis (for cases) or the matched case's age at diagnosis (for controls). Either this option or casedob(), but not both, must be specified.

**casevars** implies that for the controls the observations for the variables specified in caseddiag(), caseage() and casedob() are set to the corresponding case's values. This option is particularly useful when the data set does not contain the case-specific variables for date of diagnosis, age or date of birth and the users prefer not to create them (e.g. by using the egen command) before running audit_cc.

**scrdate(***varname***)** specifies the variable containing the date of the screening test. This option is required.

**result(***varname***)** identifies the variable containing the screening test results; *varname* must be a numeric variable with integer values indicating the following:

−1 = “adequate but not otherwise specified”

1 = “inadequate”

2 = “negative”

3 or above = “positive”

Values below −1 or equal to zero are treated as missing test results. When *varname* contains observations coded as −1 the nosresults option must be specified, in that case the analysis for the time-since-last-negative-test exposure is not performed. The result() option is required.

**agecutpoints(***numlist***)** specifies the cut-points (in years) to be used for the age stratification. For example, agecutpoints(25 40 55 70) tells Stata to display age-specific estimates for the 3 age groups: [25,40), [40,55) and [55,70), where here a square bracket means that the value is included whereas a round bracket means it is excluded (e.g. [40,55) refers to the interval 40≤age<55). The matched groups where the case's age is below the minimum or ≥ the maximum cut-point (i.e. age<25 or age≥70 in the above example) are excluded from the analysis. The choice of the age cut-points could reflect the different screening guidelines in force depending on the woman's age. The default in audit_cc is agecutpoints(25 50 65 100).

**tlcutpoints(***numlist***)** specifies the cut-points (in years) to be used for the two screening exposure measures, i.e. time since the last test and time since the last negative test. The cut-points define time intervals, with the last one being an open interval. For example, tlcutpoints(0 1 3 5) leads to four intervals: [0,1), [1,3), [3,5) and [5,+∞). Note that the last cut-point is the lower bound of the last interval. Users interested in an “ever having had a test” exposure could, for instance, use tlcutpoints(0 150). The default is tclcutpoints(0 0.5 3.5 5.5 9.5).

**minage(***#***)** indicates the age (in years) at first invitation for screening. All screening tests carried out before the age specified in minage() are ignored. The default is minage(25).

**maxage(***#***)** indicates the age (in years) at last invitation for screening. All screening tests carried out after the age specified in maxage() are ignored. The default is maxage(100).

**anythreshold(**#**)** specifies the exclusion period (in months) related to the case's occult invasive phase when deriving the time-since-last-test exposure. All screening tests carried out during that period are disregarded in the analysis for time since last test. For example, anythreshold(12) indicates a 12-month exclusion period. The default is anythreshold(6).

**negthreshold(**#**)** specifies the exclusion period (in months) related to the case's occult invasive phase when deriving the time-since-last-negative-test exposure. All negative tests carried out during that period are disregarded in the analysis for time since last negative test. The default is negthreshold(0), i.e. no exclusion period.

**nosresults** must be specified when the variable in result() contains test results coded as −1 (i.e. “adequate but not otherwise specified”). When this option is used, the analysis for the time-since-last-negative-test exposure is omitted.

**coefficients** reports the estimated coefficients rather than the odds ratios (exponentiated coefficients). This option affects only how results are displayed and not how they are estimated.

**nodetail** suppresses part of the output in the Results windows and in the saved file if saving() is specified. The tables displaying the number of observations retained in each age-specific estimation are omitted.

**saving(***filename*[, replace]**)** saves the results in an external file. If *filename* is specified without extension, .docx is assumed and the results are saved in a Word document. Possible file formats are .docx, .log and .smcl. If the sub-option replace is supplied, Stata overwrites the file in case it already exists.

**data(***filename*[, replace]**)** saves a data set containing the records (one per woman) used to fit the age-specific conditional logistic models. If *filename* has no file extension, .dta is assumed. If the sub-option replace is specified, Stata overwrites the file in case it already exists. The saved data set contains the variables specified in groupid() and id() along with agegroup (categorical age variable created using the cut-points in agecutpoints()), the two exposure measures (Time_since_last_test and Last_screened_negative), weights (if weights are supplied). The variable Last_screened_negative is omitted when the nosresults option is specified.

**noheader** has an effect only when the results are saved in a file using the saving() option. It suppresses the header information (name of the command, Stata version and current date) at the top of the Word, log or smcl document.

## 4 Examples

To illustrate the use of the audit_cc command we created artificial data resembling real cervical cancer screening records. Specifically, we generated a sample of 3,000 women (cases) diagnosed with cervical cancer between 2015 and 2020. For simplicity, we included 2,000 cases aged 20 to <50 years, 500 aged 50 to <65 and 500 aged 65 or over. Each case was then matched by age (this was an approximate matching with age within a ±2-year range) and geographical area with 2 controls. For both cases and controls we generated screening histories and corresponding test results. A description of the variables contained in this simulated dataset (*auditdata.dta*) is reported in Table 1.

**Table 1:** Variables in *auditdata.dta*.

Variable name	Description
matchgrp	unique identifier for the matched groups
woman_id	unique identifier for the women
case	type of case or control (1 = “case”, 2 or 3 = “control”)
case_ddiag	date of diagnosis of the case in the matched group
case_dbirth	date of birth of the case in the matched group
case_age	age at diagnosis of the case in the matched group
testdate	date of the screening test
testresult	result of the screening test (1 = “inadequate”, 2 = “negative”, 3 = “low-grade” and 4 = “high-grade”)

The dataset is in long format, i.e. each record corresponds to a separate screening test. Women who had no screening test during the study period are included in the dataset by recording a test entry with a missing value (.) on both testdate and testresult.

### 4.1 Example 1

Let's consider a simple scenario in which we want the results to be stratified by two age groups (25 to <50 and 50 to <65 years) and we use the tlcutpoints() option to specify cut-points for the screening exposures.

**Figure:**
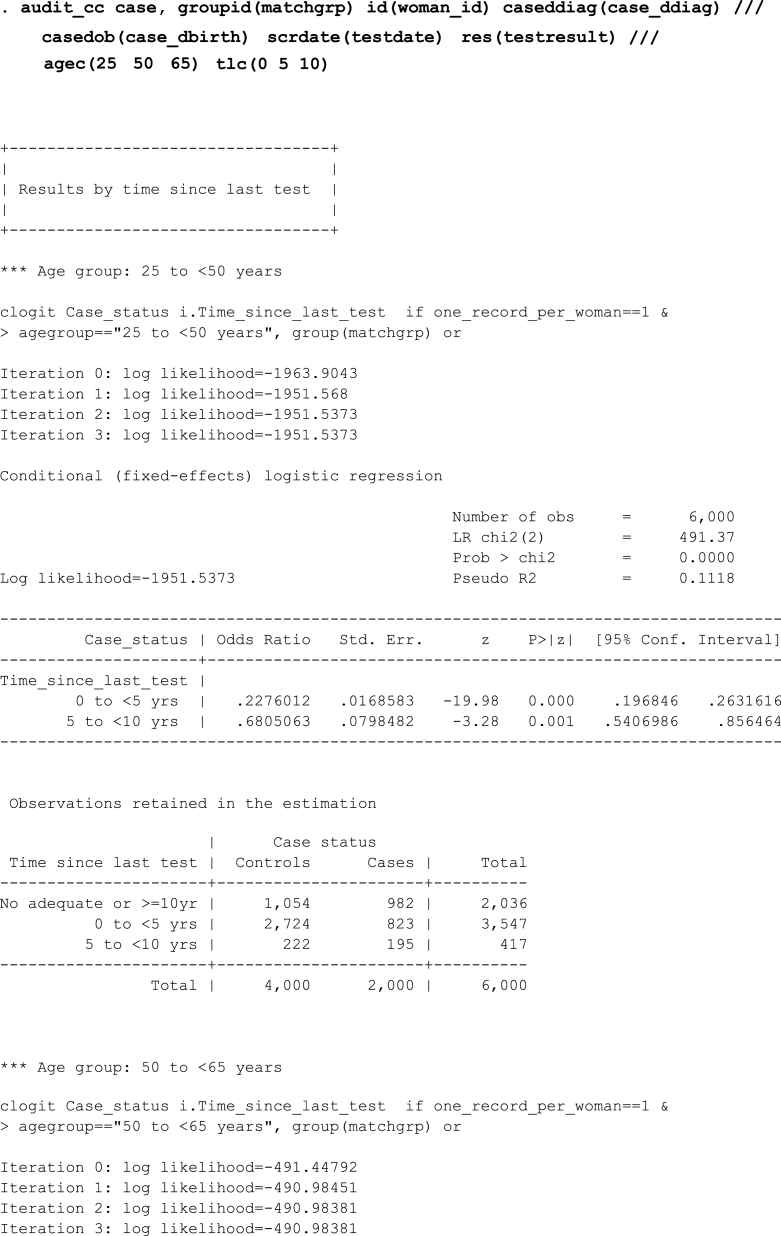

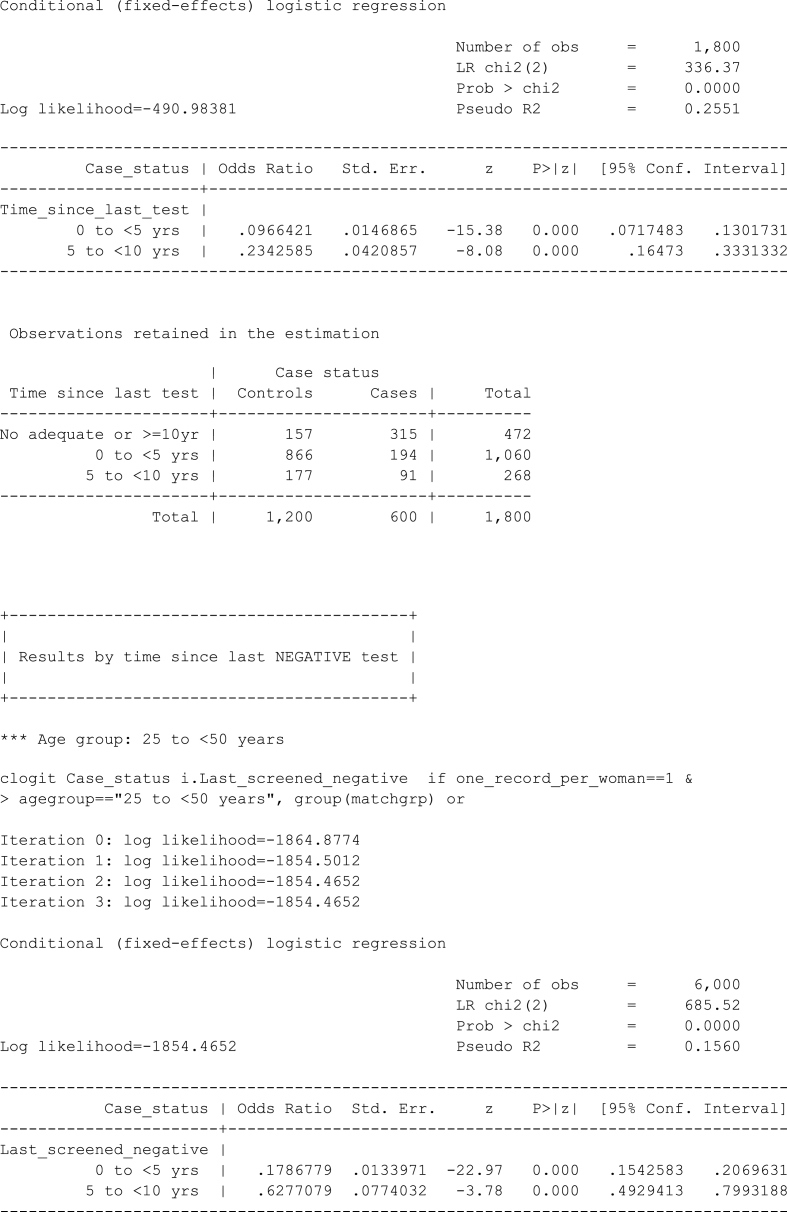

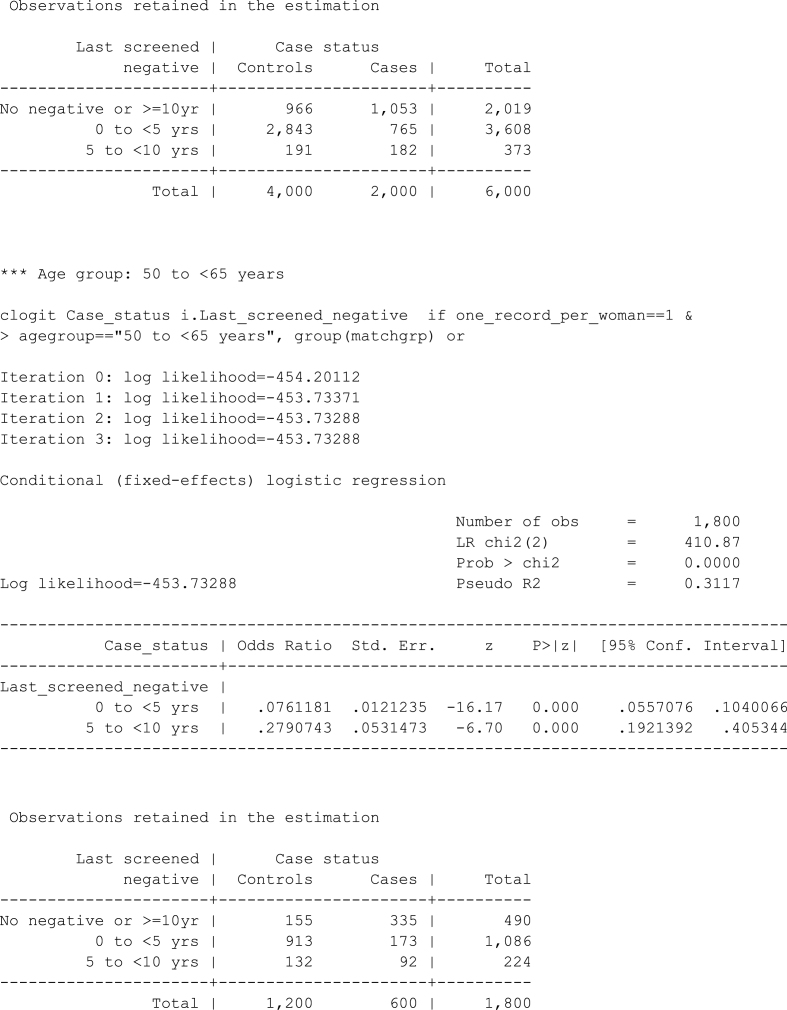

Since the default cut-points for age are 25, 50, 65 and 100, the above output could also be obtained by specifying an if qualifier instead of using the agecutpoints() option.

**Figure:**
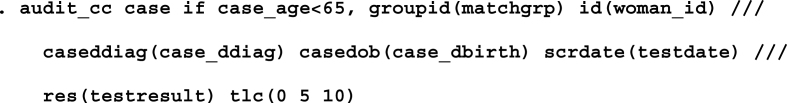

The if qualifier is also useful when one wants to carry out further sub-group analyses.

### 4.2 Example 2

In this example we use the default settings for the age groups and the screening exposure cut-points but we restrict the analysis to age<50 years and we exclude all the screening tests performed during the 9 months (the default is 6 months) prior to the case's data of diagnosis. We also want to save the results in a Word document called *myresults* in “*C:\CervicalScreening*” (the file already exists so we specify the sub-option replace in saving()). In addition, we use the noheader option to suppress part of the output and we set showbaselevels to on so that the reference categories are displayed in the result tables.

**Figure:**
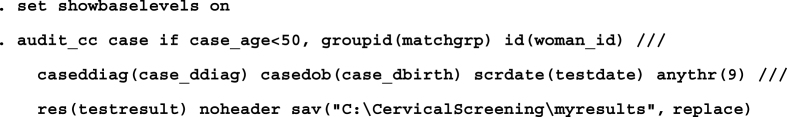

**Fig. 1:**
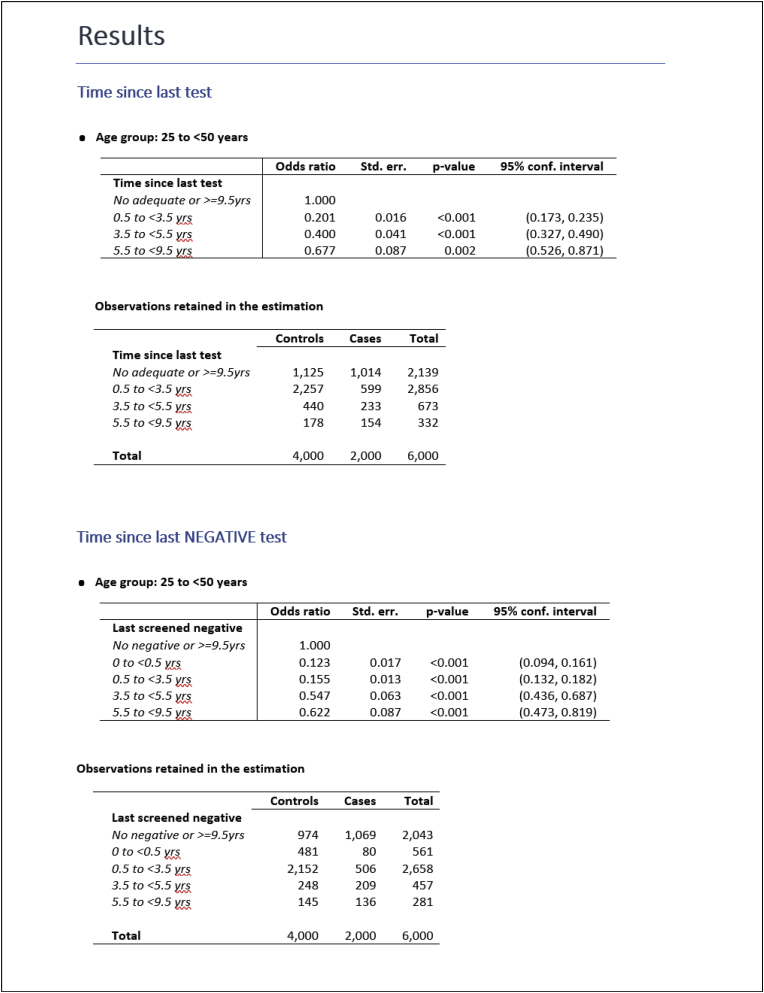
Content of the *myresults.docx* file.

The *myresults.docx* file can be opened by double clicking on it in the folder it was saved in or by clicking on the hyperlink at the very end of the on-screen output. The content of the file is displayed in Fig. 1.

In the previous command we could have also specified the data() option and saved the dataset used for the estimation of the age-specific conditional logistic models in e.g. *mydata.dta*. This would be useful if, for example, we want to use post-estimation commands (e.g. test or margins) or to identify which observations were dropped by a specific clogit model (i.e. we need to access the information stored in e(sample)). The dataset would contain all the variables we need to re-fit a specific model of interest. If for instance we want to reproduce the results of the first table in Fig. 1, that is the model for agegroup = 1 (i.e. “25 to <50”) and the time-since-last-test exposure, then we would open *mydata.dta* and type

**Figure:**
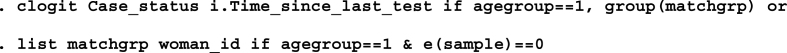

Saving the dataset with the data() option can also be very convenient when further analyses are needed. For example, users might want to fit models with additional covariates to adjust for confounding.

## 5 Conclusions

In this article we have presented a new command that facilitates the analysis and reporting of matched case-control audits of cervical screening programmes. It uses standard conditional logistic regression to account for the matching but offers several advantages.

1. The creation of two widely used screening history exposure measures: time since last test and time since last negative test. In addition, the “ever having been screened” exposure can be specified as a special case of the former.
2. The data manipulation required for the analysis and the possibility to save the resulting data set in an external file for further investigations.
3. A particular convenient and easy way to create automatic reports with results displayed in publication-quality tables.
4. It allows consistent estimates over time and across studies and facilitates international comparisons.

Although our command is specific for cervical cancer, it can also be seen as an example of how the standardization of exposure definitions and key methodological issues can enable meaningful and consistent comparisons of screening programmes across different countries and settings.

## Funding

This work was supported by 10.13039/501100000289Cancer Research UK (grant number: C8162/A27047).

## Declaration of competing interest

The authors declare that they have no known competing financial interests or personal relationships that could have appeared to influence the work reported in this paper.

## References

[1] Sasieni P., Cuzick J. (2001). Routine audit is an ethical requirement of screening. BMJ.

[2] Cuzick J. (2008). Routine audit of large-scale cervical cancer screening programs. J Natl Cancer Inst.

[3] Weiss N.S. (1994). Application of the case-control method in the evaluation of screening. Epidemiol Rev.

[4] Public Health England (2019). Cervical screening: invasive cervical cancer audit 2013 to 2016. https://www.gov.uk/government/publications/cervical-screening-invasive-cervical-cancer-audit-2013-to-2016/].

[5] Castañon A., Kamineni A., Elfstrom K.M., Lim A.W.W., Sasieni P. (2021). Exposure definition in case-control studies of cervical cancer screening: a systematic literature review. Cancer Epidemiol Biomarkers Prev.

[6] StataCorp (2021).

[7] Breslow N.E., Day N.E. (1980).

[8] Hosmer D.W., Lemeshow S.A., Sturdivant R.X. (2013).

[9] Gould W. (2000). Interpreting logistic regression in all its forms. Stata Technical Bulletin.

[10] Wan F., Colditz G.A., Sutcliffe S. (2021). Matched versus unmatched analysis of matched case-control studies. Am J Epidemiol.

[11] Weiss N.S. (1999). Case-control studies of the efficacy of screening tests designed to prevent the incidence of cancer. Am J Epidemiol.

[12] Sasieni P., Cuzick J., Lynch-Farmery E. (1996). Estimating the efficacy of screening by auditing smear histories of women with and without cervical cancer. The National Co-ordinating Network for Cervical Screening Working Group. Br J Cancer.

